# Food assistance is associated with improved body mass index, food security and attendance at clinic in an HIV program in central Haiti: a prospective observational cohort study

**DOI:** 10.1186/1742-6405-7-33

**Published:** 2010-08-26

**Authors:** Louise C Ivers, Yuchiao Chang, J Gregory Jerome, Kenneth A Freedberg

**Affiliations:** 1 Division of Global Health Equity, Brigham and Women's Hospital, Boston, Massachusetts, USA; 2 Partners In Health, Boston, Massachusetts, USA; 3 Biostatistics and Computational Biology, Harvard University Center for AIDS Research, Cambridge, Massachusetts, USA; 4 General Medicine Division, Massachusetts General Hospital, Boston, Massachusetts, USA; 5 Zanmi Lasante, Cange, Haiti

## Abstract

**Background:**

Few data are available to guide programmatic solutions to the overlapping problems of undernutrition and HIV infection. We evaluated the impact of food assistance on patient outcomes in a comprehensive HIV program in central Haiti in a prospective observational cohort study.

**Methods:**

Adults with HIV infection were eligible for monthly food rations if they had any one of: tuberculosis, body mass index (BMI) <18.5kg/m^2^, CD4 cell count <350/mm^3 ^(in the prior 3 months) or severe socio-economic conditions. A total of 600 individuals (300 eligible and 300 ineligible for food assistance) were interviewed before rations were distributed, at 6 months and at 12 months. Data collected included demographics, BMI and food insecurity score (range 0 - 20).

**Results:**

At 6- and 12-month time-points, 488 and 340 subjects were eligible for analysis. Multivariable analysis demonstrated that at 6 months, food security significantly improved in those who received food assistance versus who did not (-3.55 vs -0.16; P < 0.0001); BMI decreased significantly less in the food assistance group than in the non-food group (-0.20 vs -0.66; P = 0.020). At 12 months, food assistance was associated with improved food security (-3.49 vs -1.89, P = 0.011) and BMI (0.22 vs -0.67, P = 0.036). Food assistance was associated with improved adherence to monthly clinic visits at both 6 (P < 0.001) and 12 months (P = 0.033).

**Conclusions:**

Food assistance was associated with improved food security, increased BMI, and improved adherence to clinic visits at 6 and 12 months among people living with HIV in Haiti and should be part of routine care where HIV and food insecurity overlap.

## Introduction

Food insecurity and undernutrition are increasingly recognized as factors that are important in the health and livelihoods of individuals living with HIV infection in poor settings [1,2]. HIV infection has long been associated with wasting syndrome and being underweight with HIV is predictive of a poor prognosis, even in people receiving antiretroviral therapy (ART) [1,3-5]. Food insecurity--meaning lack of access to food of sufficient quality and quantity to perform usual daily activities--contributes to a negative cycle of events that often worsens the effect of HIV infection on ability to work, attend school, contribute to family livelihoods and adhere to medications [6-8]. International organizations have called for food assistance to be integrated into HIV treatment and prevention programs, but evidence-based guidance on how exactly to implement such programs, on what beneficiaries to target, and on what the optimal components or duration of food assistance should be is limited [9-14]. A recent study showed that food rations were associated with improved adherence to ART, but these data did not show any quantitative clinical benefit [15].

Attention to adequate nutrition during HIV care has the potential to contribute to improved clinical HIV-related outcomes, improved nutritional outcomes for the individual, as well as improved coping strategies and ability of individuals to contribute to livelihoods at the household level. Although the qualitative effect of food on relieving hunger is not in doubt, the quantitative benefits of food assistance on individuals or on families has rarely been studied in the context of HIV [16]. As a result of political instability, environmental degradation, poverty and recurrent natural disasters, Haiti is extremely vulnerable to food insecurity. The aim of this study was to determine the impact of targeted food assistance on the body mass index (BMI), quality of life and household food security of people living with HIV in a comprehensive health program in central Haiti.

## Methods

The study was a prospective observational cohort study of 600 people living with HIV enrolled in HIV care in Partners In Health (PIH) programs in rural Haiti. PIH is a non-profit organization working in conjunction with the Ministry of Health of Haiti to provide comprehensive primary healthcare services, including HIV care, in two departments in rural Haiti. In May 2006, PIH entered into collaboration with the World Food Programme (WFP) to provide food rations for beneficiaries living with HIV. Because available rations were limited, beneficiaries of the program were determined by a set of criteria agreed upon in advance by WFP and PIH program staff, including clinicians, social workers, and ethicists. Adults received twelve months of food assistance if they had HIV and any one of: co-infection with active TB, CD4 count less than 350 cells/mm^3 ^in the prior three months, BMI less than 18.5 or severe socioeconomic circumstances (based on social worker assessment and clinical team consensus). A standard pre-determined WFP family ration was provided by prescription monthly. The ration contained 50 gm of cereal, 50 gm of dried legumes, 25 gm of vegetable oil, 100 gm of corn-soya blend and 5 gm of iodized salt for each of 3 family members (approximately 949 kilocalories) per person per day.

Three PIH clinic sites were included in the study (one rural, one urban, one semi-urban). At each site the first 100 individuals eligible for food assistance and first 100 ineligible for food assistance by the criteria defined above were invited to participate in the study. Individuals were eligible for interview if they were living with HIV, were being assessed by the clinical team for eligibility for the food program, were over the age of 18 years and were not pregnant at the time of interview.

Combination ART is offered to those with HIV infection and CD4 counts less than 350 cells/mm^3 ^or with World Health Organization clinical criteria to begin treatment. Pregnant women are offered ART for their own health when CD4 count is less than 350 cells/mm^3 ^or at 28 weeks of gestation for prevention of mother-to-child transmission. Weight is measured routinely during patient monthly visits to clinic. Height was measured for adults at the beginning of the WFP collaboration using a clinic-installed stadiometer to allow calculation of BMI by clinic staff. In addition to medical care, attention is paid to the socioeconomic causes and contributors to disease and ill-health, and social assistance programs make small grants for commerce or housing repair available. All care is provided free of charge to patients [17].

### Surveys

Individuals were interviewed before rations were distributed and at 6 months and 12 months after food assistance began. Data collected in surveys included demographics, education level, BMI, food insecurity score and quality of life. Additional information was abstracted from the respondent's electronic medical record including CD4 count, timely attendance at prescribed monthly clinic visits, weight, BMI and pick up of prescribed food rations. Food insecurity score was measured using the Household Food Insecurity Access Scale (HFIAS) [18]. In this scale (ranging from 0 for best food security to 20 for worst), points are attributed for items that relate to the availability of food in the household. The authors had previously refined and adapted this questionnaire for use in rural Haiti using the methods recommended by Coates et al [18,19]. Quality of life was measured using role-functioning and performance-functioning domains adapted from the AIDS Clinical Trials Group SF-2, with scores ranging from 0 to 100 [20]. Instruments were translated into Haitian Creole and back-translated to English for accuracy. Interviews were performed by native Haitian Creole speakers. Data were double-punch entered into MS Access database. Ethics committee approval was received for the study from the Zanmi Lasante (Partners In Health) Ethics Committee in Haiti and by the Institutional Review Board at Brigham and Women's Hospital, Boston, MA, USA.

### Analysis

Duration on ART was determined for each respondent at the time of entry into the study and the respondent analyzed in this category throughout the study: 'never on ART', 'on ART < 12 months, 'on ART ≥12 months'. Active TB infection was an absolute indication for receiving food assistance in the PIH program. Since no active TB patient would be found in the 'non-intervention' group, and because TB contributes to weight loss independently of HIV infection, subjects that had active TB during the period of the study were excluded from the final analysis. Subjects that were either enrolled in or discontinued from food rations by the clinical team during the period of the study were also excluded from the final analysis ('as-treated analysis'). Individuals were also excluded from analysis if they became pregnant during the study. BMI analysis was limited to those with weight available (N = 4). We also performed a sensitivity analysis using an 'intention to treat' approach, including all subjects based on their enrollment status at the time of baseline evaluation. For those with missing food security items, the response was replaced by the median value from all respondents in the same phase of the study (i.e. baseline, 6 months or 12 months). We also performed a sensitivity analysis using the E-M algorithm to impute missing food security items at 6 and 12 months.

Baseline data were summarized using mean/standard deviation (SD) or percentage and compared between the 'no food assistance' group and the 'food assistance' group using two-sample t-tests or Chi-square tests. Continuous outcomes of change from baseline were summarized using mean/standard error (SE). In the univariate analysis, Wilcoxon rank sum tests were used to compare continuous outcomes while repeated measures logistic regression with Generalized Estimating Equations (GEEs) were used to compare dichotomized outcomes. In the multivariable analysis, linear regression and repeated measures logistic regression analysis were used to compare the change from baseline between the two groups controlling for other factors. All analyses were performed using SAS version 9.2 (SAS Institute, Cary, NC).

## Results

**Figure 1:**
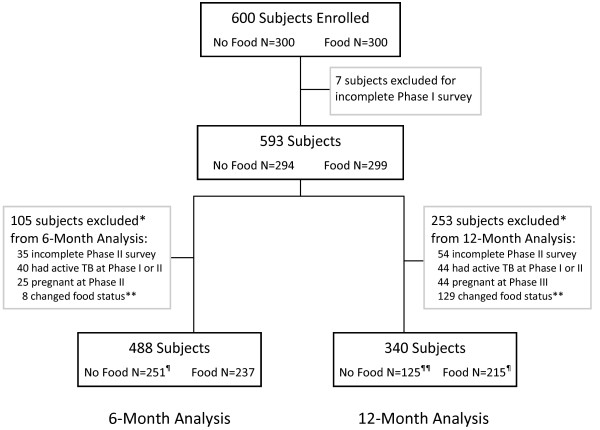
**Distribution of subjects with HIV enrolled in observational cohort study in central Haiti**. TB = tuberculosis. BMI = body mass index. * not mutually exclusive. ** all changes in food status were from "No Food" to "Food Assistance" status. ¶ 1 subject did not have BMI data and was not included in BMI analysis. ¶¶ 3 subjects did not have BMI data and were not included in BMI analysis

Between May and July 2006, 600 adults were enrolled across the three clinical sites. At 6- and 12-month follow ups, 488 and 340 individuals were eligible for the analysis (Figure 1).

### Baseline Characteristics

The 488 adults in the study who were eligible for 6-month follow up had mean (SD) age of 36 years (10); 60% were female (Table 1). The majority of participants (71.7%) spent all or most of their monthly income on food. At baseline, 148 (30.3%) were not on antiretroviral therapy (ART), 279 (57.2%) had been on ART for ≥12 months, and 61 (12.5%) had been on ART for <12 months. At baseline, compared to the group that did receive food assistance, the group receiving no food assistance contained fewer individuals on ART [N = 145 (57.8%) vs. N = 195 (82.3%), P < 0.0001], had better food security (13.9 vs. 15.4, P < 0.0001), had higher BMI (22.4 vs. 20.4, P < 0.0001) and had more individuals sharing household meals on average (6.7 vs.6.1, P = 0.035). Similar patterns persisted in the assessment of the 340 subjects eligible for 12-month follow up.

**Table 1:** Baseline characteristics at 6 and 12 months of a cohort of people with HIV in central Haiti

	Subjects with 6-month follow-up					Subjects with 12-month follow-up				
		
	No Food group (N = 251)		Food Assistance group (N = 237)		P value	No Food group (N = 125)		Food Assistance group (N = 215)		P value
Age, mean yrs (SD)	35	(9)	37	(10)	0.12	36	(10)	37	(10)	0.26
Female, N (%)	149	59.4%	144	60.8%	0.75	74	59.2%	126	58.6%	0.91
Proportion of monthly income spent on food*, N (%)					0.80					0.68
None	22	8.8%	28	11.8%	_	10	8.0%	25	11.6%	_
Small amount	13	5.2%	10	4.2%	_	7	5.6%	8	3.7%	_
Half	28	11.2%	29	12.2%	_	13	10.4%	27	12.6%	_
Most	72	28.7%	65	27.4%	_	38	30.4%	58	27.0%	_
All	112	44.6%	101	42.6%	_	55	44.0%	94	43.7%	_
ART group, N (%)					<0.0001					<0.0001
No ART	106	42.2%	42	17.7%	_	47	37.6%	40	18.6%	_
On ART ≥ 1 yr	134	53.4%	145	61.2%	_	69	55.2%	130	60.5%	_
On ART < 1 yr	11	4.4%	50	21.1%	_	9	7.2%	45	20.9%	_
Female-headed household*, N (%)	123	49.0%	112	47.3%	0.67	63	50.4%	102	47.4%	0.58
Literate*, N (%)	148	59.0%	127	53.6%	0.25	75	60.0%	119	55.3%	0.43
Number sharing household meals, mean (SD)	6.7	(2.9)	6.1	(2.9)	0.035	6.8	(2.9)	6.3	(2.9)	0.18
Food insecurity score**, mean (SD)	13.9	(3.9)	15.4	(3.9)	<0.0001	14.0	(4.1)	15.3	(3.9)	0.003
Body mass index†, mean kg/m^2 ^(SD)	22.4	(2.7)	20.4	(3.2)	<0.0001	22.5	(3.0)	20.2	(3.0)	<0.0001

SD = standard deviation

ART = antiretroviral therapy

* sample size varies due to missing survey responses

** range 0 (best food security) to 20 (worst food insecurity

† Body mass index range: ≤18.5 = underweight; 18.5-24.9 = normal; ≥25.0 = overweight

### Food Insecurity and Body Mass Index

In univariate analysis at 6 and 12 months, food security was improved in the group that received food compared to the non-food group (Table 2). On a scale of 0 (best) to 20 (worst), mean change (SD) in food insecurity score in the food assistance group was -3.55 compared to -0.16 in the non-food group at 6 months (P < 0.0001) and -3.49 compared to -1.89 at 12 months (P = 0.011). At 6 months, BMI decreased in both groups, but fell less in the food assistance group compared to the non-food group (-0.20 vs. -0.66, P = 0.012). At 12 months, BMI increased in the food group and decreased in the non-food group (+0.22 vs. -0.67, P = 0.002).

**Table 2:** 6-month and 12-month outcomes among a cohort of people living with HIV in Haiti who did and did not receive food assistance

	6-month outcomes						12-month outcomes					
		
	No food group (N = 251)		Food Assistance group (N = 237)		Univariate P value	Multivariable P value*	No food group (N = 125)		Food Assistance group (N = 122)		Univariate P value	Multivariable P value*
Change in Food Insecurity Score, mean (SE)	-0.16	(0.28)	-3.55	(0.33)	<0.0001	<0.0001	-1.89	(0.47)	-3.49	(0.33)	0.011	0.011
Change in body mass index**, mean (SE)	-0.66	(0.13)	-0.20	(0.13)	0.012	0.020	-0.67	(0.22)	0.22	(0.17)	0.002	0.036
Adherence to scheduled monthly clinic visits, mean number attended (SE)	2.82	(0.20)	5.49	(0.17)	<0.0001	<0.0001	8.34	(0.44)	9.73	(0.32)	0.007	0.033
Change in QOL (role-functioning), mean (SE)	-5.08	(3.01)	-1.90	(2.98)	0.28	0.84	-3.80	(4.03)	3.72	(3.46)	0.13	0.11
Change in QOL (performance-functioning), mean (SE)	5.31	(1.77)	10.69	(1.99)	0.055	0.009	9.47	(2.67)	8.76	(2.17)	0.48	0.69
Reports problem taking ART^†^, N (%)	41	28.1%	26	14.4%	0.001	0.001	15	18.8%	18	11.0%	0.068	0.075
Was able to save money in case of disaster**, N (%)	66	26.5%	45	19.1%	0.63	0.35	41	33.3%	52	24.9%	0.59	0.42
Spent less money on agriculture to buy food**, N (%)	75	38.1%	57	31.7%	0.58	0.57	37	39.8%	48	25.9%	0.11	0.082
Spent less on education to buy food**, N (%)	84	36.5%	75	33.9%	0.75	0.63	34	30.6%	68	33.7%	0.28	0.25
Sold livestock to buy food^‡^, N (%)	43	41.3%	25	24.5%	0.092	0.082	20	38.5%	25	25.0%	0.097	0.11

SE = standard error

ART = antiretroviral therapy

QOL = quality of life

* Controlling for gender, literacy, ART group, number of people sharing meals in the household

** Sample size varied due to missing survey responses

^† ^Limited to those who were on ART

^‡ ^Limited to those who owned livestock

### Adherence to clinic visits and medications

At both 6 and 12 months, timely attendance at monthly clinic visits was better in the food assistance group than in the non-food group. The mean number of scheduled visits attended at 6 months (out of 6 visits) was 5.49 vs. 2.82 (P < 0.0001) for the food assistance vs. the non-food group, and at 12 months (out of 12 visits) was 9.73 vs. 8.34 (P = 0.007).

### Quality of life

There was no statistical difference in role-functioning quality of life (QOL) between the groups at 6 months. At 12 months, mean role-functioning QOL score increased in the food assistance group (3.72) and decreased in the non-food group (-3.80), however the difference did not reach significance level (P = 0.13). Performance-functioning QOL had a slightly greater increase at 6 months in the food assistance group compared to the non-food group (mean change 10.69 vs. 5.31, P = 0.055). There was no difference at 12 months between the two groups (8.76 vs.9.47, P = 0.48).

Among those on ART, at 6 months, those receiving food assistance reported fewer difficulties taking their medications compared to those who did not receive food (14.4% vs. 28.1%, P = 0.001). At 12 months, although a difference remained between the groups (11.0% vs. 18.8%), it did not reach statistical significance level (P = 0.068). There were no significant differences between groups at either 6 or 12 months in terms of ability to save money in case of disaster, spending on agriculture or education, or spending on livestock. There was also no statistical difference in outcomes of died or abandoned care at 6 or 12 months.

There were 129 individuals who changed from the non-food group to the food assistance group during the course of the study. Most (N = 121) of these changes occurred during the second half of the study in the 6-to-12-month period that coincided with the 'lean season' in Haiti. It is possible that individual's socioeconomic status worsened during this time, prompting the team to enroll individuals in food assistance based on these criteria. In addition, as the program timeline progressed, rations that had not yet been assigned within the total number of program rations available may have prompted an informal relaxation of the socioeconomic criteria for eligibility. Of the 129 individuals that switched status from 'no food assistance' to 'food assistance', seven had BMI < 18.5 and eight had CD4 count < 350 cells/mm^3 ^at the 6-month evaluation; one had TB and 12 were pregnant at the 12-month survey. When compared to the 125 individuals that did not change status from 'no food' to 'food assistance', they had slightly less improvement in food security (-0.10 vs. -0.20) and slightly worse BMI (-0.72 vs. -0.61) at 6 months, but neither was significant (P = 0.81, P = 0.97). These individuals were excluded from the final analysis (Figure 1).

### Multivariable analysis

In establishing the multiple regression analysis model we used existing literature, including the conceptual framework of Egge et al [1,2,8,21] to establish factors of importance a priori. We also accounted for the baseline differences between the two groups. The final model compared the two groups controlling for gender, literacy, ART group and number of people sharing meals in the household. At both 6 and 12 months, food assistance was associated with better food security (P < 0.0001 and P = 0.011), improved BMI (P = 0.020, P = 0.036), better adherence to monthly clinic visits (P < 0.0001, P = 0.033) compared to no food assistance. A sensitivity analysis including all patients with tuberculosis did not change the outcome of the study.

## Discussion

This study finds that providing food assistance to individuals with HIV and food insecurity in central Haiti improves BMI, food security and adherence to clinic visits. We also observed a significant improvement in ability to take ART at 6 months and a trend for improvement in this variable at 12 months. Although many studies have evaluated the impact of micronutrient supplementation on HIV disease progression, to our knowledge, this is the first study demonstrating a quantitative clinical benefit of macronutrient supplementation on HIV clinical outcomes.

Low BMI may result from chronic inadequate food intake, wasting as a result of HIV or other infections, or in some individuals may be a normal anthropometric variation. Low BMI has previously been independently associated with poor outcomes among individuals with HIV infection even while on ART [22-24]. In this study, after 6 months, there was a decrease in BMI in both groups, although the group not receiving food assistance had significantly greater decrease in their BMI. At 12 months, loss in body mass persisted in the no food assistance group, but BMI had increased significantly in the group receiving food assistance. The 6-month evaluation was conducted during the dry season, which is the typical 'lean season' in central Haiti, with food usually more scarce. It is likely that this contributed to both groups losing weight at that time.

Of note, the pre-determined WFP ration distributed to support people living with HIV in Haiti provides approximately 45% of the caloric requirement of a family of three, although the median number of people eating at the households in this study was six. Furthermore, the ration is not targeted specifically to individuals with HIV infection, but is rather a family support. Despite these issues, that by virtue of quantity and quality might be expected to attenuate the effect of food support on the individuals we studied, food rations were protective against weight loss in the short-term and associated with weight gain in the long-term for the individuals with HIV.

Seventy-two percent of the participants in this study spent most or all of their income on food and mean baseline food insecurity was very high (14.6 on a scale of 0 to 20). This is consistent with national statistics for the region [25]. In our study, food rations for people living with HIV were associated with significant improvements in food security at both 6 and 12 months. In addition to relief of anxiety regarding food availability, the programmatic importance of improving food security can be considered in terms of its effects on general health, nutrition, HIV infection and health services usage. The negative interactions between food insecurity and HIV are well known [5,8,26-28]. In Canada food insecurity was a risk factor for mortality among individuals with HIV on ART, particularly when this was associated with being underweight [29]. Food insecurity was also associated with incomplete viral suppression among HIV-infected urban poor in San Francisco [30]. In non-HIV infected individuals, food insecurity has been associated with self-reported poor health and depressive symptoms [31], with postponing needed medical care and high rates of emergency department usage [32] and as a strong predictor of symptoms of anxiety and depression [33]. In Haiti, household food insecurity has recently been associated with childhood malaria [34]. Interventions that result in quantitative improvements in food security, as found in our study, have potentially broad-reaching implications for the health of people living with HIV.

This current study also found that individuals receiving food assistance were significantly more likely to attend scheduled clinic visits than those not receiving food assistance. Loss to follow up of individuals not yet on ART, and those on ART, is a critical challenge to HIV care in resource-poor countries. Studies have shown early loss to follow up of as many as 21% of individuals newly started on ART at 6 months [35,36]. In a fee-for-service clinic in urban South Africa, 16% of individuals eligible for ART were lost to follow up even before ART could be started [37]. In our program, people living with HIV are provided a transportation stipend to attend monthly clinic visits. This is intended to offset their opportunity costs for the visit, thus encouraging attendance and, along with a comprehensive package of services free of charge to patients, contributes to an improved level of attendance and a very low rate of loss to follow up over time [38]. Despite the already good baseline attendance, however, we found that food assistance is an important factor in keeping food insecure individuals with HIV infection engaged in care.

Lack of sufficient food in the diet has further negative effects on HIV care by impacting ability to take antiretroviral medications in a number of ways, including causing symptoms of nausea while taking medications on an empty stomach, increasing drug toxicity, and/or forcing competing choices between expenditures on food and transportation or other health-related needs. Cantrell and colleagues demonstrated that food supplementation was associated with improved adherence to ART in an HIV clinic in Zambia [15], but that study failed to demonstrate a direct benefit of the food supplement on clinical outcomes--potentially because of lack of power to detect a difference. This study found that food assistance was associated with fewer difficulties in taking medications. This finding was statistically significant at 6 months, but not at 12 months, although the 12-month trend was towards a benefit of food assistance. This is an important finding, given that very high levels of adherence to ART are necessary for viral suppression and the subsequent benefits of ART on the health of individuals with HIV, and that adherence to ART is a powerful predictor of survival among people living with HIV [36].

HIV program managers had flexibility to enroll individuals in food assistance throughout the period of this study because the study was observational. This happened in particular between months 6 and 12 of the study as WFP made an increased number of rations available and evaluation of socioeconomic status became more inclusive. In order to examine the food rations effect in an "uncontaminated" fashion, we focused our analysis on comparing the group of subjects that were eligible for and remained in the food assistance program from baseline to the group of subjects that were never eligible for food assistance. Subjects not receiving rations at baseline but who were enrolled for rations during the study (and vice versa) were excluded from the analysis ('as-treated' analysis). We also performed a sensitivity analysis that was 'intention to treat', including subjects based on their enrollment status at the time of baseline evaluation. This analysis demonstrated that at 6 and 12 months food assistance was associated with improvements in food security (P < 0.001 and P = 0.03) and better adherence to clinic visits (P < 0.0001 and P < 0.0001) compared to no food assistance. There were trends towards difference in BMI similar to those found in the as-treated analysis, but these did not have statistical significance (P = 0.07 and P = 0.16). A further sensitivity analysis was performed using an alternate method for imputing missing data. Using the E-M algorithm at 6 and 12 months [39], food assistance was associated with better BMI (P = 0.020 and P = 0.036) and better adherence to clinic visits (P < 0.0001 and P = 0.03) compared to no food assistance. Food security was better at 6 months (P = 0.003) in the food assistance group compared to the no food group. At 12 months there was a trend for improvement in food security, but this was not significant (P = 0.12). The E-M method of imputation reflects the uncertainty of missing data and increases variability compared to using median values, thereby likely reduced power to detect differences in the data[39].

### Study Limitations

The study has several limitations. It was an observational study and subjects were not randomly selected to receive the intervention. We attempted to control for measured differences between the groups using multivariable analysis however other factors not controlled in the analysis may have influenced the associations among food security, food assistance and other outcomes. With regards to unmeasured bias, subjects in the intervention group were 'worse-off' or 'more vulnerable' than the subjects in the control group by virtue of the design of the food assistance program, which aims to help those that are considered the most vulnerable. The food assistance group had lower weight, higher food insecurity and was more likely to be on ART than the no food assistance group before the study began and may have had other unmeasured differences that could have systematically influenced the outcome. Since those that are 'more vulnerable' may be expected to do worse over the course of 12 months than those in the 'less vulnerable' control group, we believe that the differences at baseline would have biased the study result to the null. If the effect we had observed was simply a phenomenon of "regression toward the mean," we would have expected the food insecurity score to be quite similar between the two groups at follow-ups. However, the food insecurity score was lower in the food group than the non-food group at both follow-up times (8.94 vs. 9.65 at 6 months and 8.95 vs. 9.16 at 12 months); therefore, the effect cannot be simply explained by regression to the mean. The fact that our study found significant differences in outcomes, despite non-randomization of the intervention, suggests that the effect of the intervention is real.

## Conclusions

This study demonstrates that food assistance is associated with improvements in clinical outcomes among people with HIV infection and food insecurity in central Haiti. Food assistance as part of comprehensive healthcare is associated with significant improvements in BMI, food security, and adherence to clinic visits at 6 and 12 months among people living with HIV. Food assistance should be standard of care in regions where HIV and food insecurity overlap.

## Abbreviations and Acronyms

ART: antiretroviral therapy; BMI: body mass index; HFIAS: Household Food Insecurity Access Scale; HIV: human immunodeficiency virus; PIH: Partners In Health; QOL: quality of life; TB: tuberculosis; SD: standard deviation; WFP: World Food Programme

## Competing interests

The authors declare that they have no competing interests.

## Authors' contributions

LCI, KAF and JGJ contributed to the design of the study, analysis of the data and to writing the manuscript. YC contributed to analysis of data and writing of the manuscript. All authors read and approved the final manuscript.
